# Preclinical characterization of tyrosine kinase inhibitor-based targeted therapies for neuroendocrine thyroid cancer

**DOI:** 10.18632/oncotarget.26480

**Published:** 2018-12-28

**Authors:** Karine Pozo, Stefan Zahler, Keisuke Ishimatsu, Angela M. Carter, Rahul Telange, Chunfeng Tan, Shuaijun Wang, Roswitha Pfragner, Junya Fujimoto, Elizabeth Gardner Grubbs, Masaya Takahashi, Sarah C. Oltmann, James A. Bibb

**Affiliations:** ^1^ Department of Neuroscience, The University of Texas Southwestern Medical Center, Dallas, TX, USA; ^2^ Department of Surgery, The University of Texas Southwestern Medical Center, Dallas, TX, USA; ^3^ Center for Drug Research, Ludwig-Maximilians-Universität, Munich, Germany; ^4^ Advanced Imaging Research Center, The University of Texas Southwestern Medical Center, Dallas, TX, USA; ^5^ Department of Surgery, The University of Alabama, Birmingham, AL, USA; ^6^ Department of Psychiatry, The University of Texas Southwestern Medical Center, Dallas, TX, USA; ^7^ Institute of Pathophysiology and Immunology, Medical University of Graz, Graz, Austria; ^8^ Department of Translational Molecular Pathology, The University of Texas MD Anderson Cancer Center, Houston, TX, USA; ^9^ Department of Surgical Oncology, The University of Texas MD Anderson Cancer Center, Houston, TX, USA; ^10^ Comprehensive Cancer Center, The University of Alabama at Birmingham Medical Center, Birmingham, AL, USA

**Keywords:** medullary thyroid carcinoma, tyrosine kinase inhibitors, vandetanib, targeted therapy, animal model

## Abstract

Medullary thyroid carcinoma (MTC) is a slow growing neuroendocrine (NE) tumor for which few treatment options are available. Its incidence is rising and mortality rates have remained unchanged for decades. Increasing the repertoire of available treatments is thus crucial to manage MTC progression. Scarcity of patient samples and of relevant animal models are two challenges that have limited the development of effective non-surgical treatments. Here we use a clinically accurate mouse model of MTC to assess the effects and mode of action of the tyrosine kinase inhibitor (TKI) Vandetanib, one of only two drugs currently available to treat MTC. Effects on tumor progression, histopathology, and tumorigenic signaling were evaluated. Vandetanib blocked MTC growth through an anti-angiogenic mechanism. Furthermore, Vandetanib had an apparent anti-angiogenic effect in a patient MTC sample. Vandetanib displayed minimal anti-proliferative effects *in vivo* and in human and mouse MTC tumor-derived cells. Based on these results, we evaluated the second-generation TKI, Nintedanib, alone and in combination with the histone deacetylase (HDAC) inhibitor, Romidepsin, as potential alternative treatments to Vandetanib. Nintedanib showed an anti-angiogenic effect while Romidepsin decreased proliferation. Mechanistically, TKIs attenuated RET-, VEGFR2- and PI3K/AKT/FOXO signaling cascades. Nintedanib alone or in combination with Romidepsin, but not Vandetanib, inhibited mTOR signaling suggesting Nintedanib may have broader anti-cancer applicability. These findings validate the MTC mouse model as a clinically relevant platform for preclinical drug testing and reveal the modes of action and limitations of TKI therapies.

## INTRODUCTION

MTC is a rare indolent cancer derived from calcitonin secreting C-cells of the thyroid gland, which occurs either as a sporadic or hereditary disease [1]. Most MTC patients carry activating mutations in the Rearranged-during-transfection (RET) gene, which encodes a receptor tyrosine kinase [2, 3]. Therefore therapeutics that target RET have been developed [4]. Two TKIs, Vandetanib and Cabozantinib, have been approved for the treatment of advanced MTC patients [5–7]. Limited drug responses as well as drug-associated adverse events have limited the number of patients for which these treatments can be used [8]. Furthermore, resistance to TKIs is common and their efficacy in stopping disease progression is not ideal [9]. Thus expanding the repertoire of therapies available for the treatment of MTC and other NE cancers is essential. In particular, defining the mode of action of current MTC drugs would advance our understanding of relevant oncogenic signaling pathways and could suggest additional drugs or combinations to more effectively treat these cancers.

Vandetanib was the first drug approved for MTC treatment in 2011 [5, 10]. It is a first generation TKI with demonstrated cross-specificity for VEGF, RET and EGF receptors [3]. However, its modes of action in MTC have not been well defined. Earlier work in cultured MTC cells as well as preclinical MTC mouse and *Drosophila* models indicated that Vandetanib impedes MTC proliferation via RET inhibition [11–13]. Paradoxically, Vandetanib-response does not correlate clearly with patient's RET-mutational status, as indicated by a randomized phase III clinical trial [5]. Overall, the efficacy of Vandetanib appears to be limited to an undefined patient sub-population. Thus insight into the mechanisms of action of Vandetanib could guide the development of more effective therapeutic strategies.

Given the limitations of the drugs currently available for clinical application, it is understandable that alternative TKI therapies are the subject of active investigation. For example, the second generation TKI, Nintedanib is a multi-kinase inhibitor that has been approved for the treatment of idiopathic pulmonary fibrosis [14] and is currently undergoing phase 2 clinical trials for MTC in Europe (ClinicalTrials.gov Identifier: NCT01788982). Nintedanib targets VEGF, FGF, and PDGF receptors [15], and blocks angiogenesis, the process by which new blood vessels form within tumors, and does not exhibit the common adverse events associated TKI. Although Nintedanib shares common targets with Vandetanib, the anti-cancer effects of these two TKIs have not previously been compared.

We have recently developed a clinically relevant mouse model of MTC in which transgenic overexpression of p25-GFP, a cyclin-dependent kinase 5 (Cdk5) activator causes MTC. Oncogenic signaling pathways that are downstream of Cdk5 and drive mouse MTC growth are also detected in sporadic MTC patients [16, 17]. In particular, p25 expression leads to inactivation of the tumor suppressor, retinoblastoma protein (Rb) and deregulated expression of cell cycle proteins, both in the mouse model tumors and in MTC patients. Here we use this animal model to characterize and compare the anti-cancer effect and modes of action of an established MTC drug, *i.e.*, Vandetanib, and that of two experimental TKI-based MTC treatments, Nintedanib alone and in combination with the HDAC inhibitor, Romidepsin, which has previously been co-administrated with TKIs.

## RESULTS

### *In vivo* monitoring of mouse tumor growth by magnetic resonance imaging (MRI)

**Figure 1:**
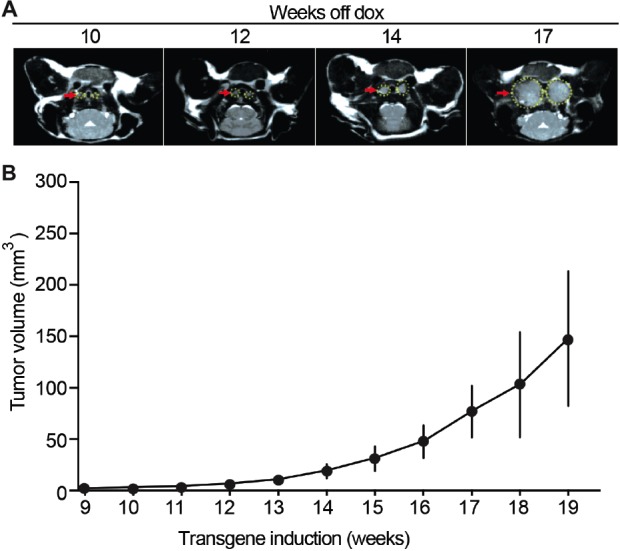
Mouse MTC tumor growth monitored *in vivo* by MRI **(A)** Representative MRI images of tumors at 10, 12, 14 and 17 weeks after transgene induction (see Methods). **(B)** Quantification of tumor volumes shows tumor growth over time after transgene induction, N = 4.

An animal model commonly used for preclinical testing of anti-cancer therapies is one in which human cells are injected subcutaneously in immunocompromised mice and tumor growth is followed manually by measurement with calipers. In contrast, NSE/p25-GFP mouse tumors are smaller and arise spontaneously within the thyroid gland, dorsal to the salivary glands [16]. Advanced *in vivo* imaging techniques such as MRI allow monitoring tumor progression in this model. To determine the optimal conditions at which to conduct preclinical drug testing, we monitored tumor volume *in vivo* by MRI starting 9 weeks after induction of p25-GFP expression (see Materials and Methods), when tumors become detectable (Figure 1A). Tumors developed from the thyroid gland as bilateral masses on each side of the trachea [16]. Initial tumor growth was slow, consistent with a neuroendocrine phenotype. Starting approximately 14 weeks after transgene induction, tumor growth accelerated and tumor volumes at least doubled over a 2-week period (Figure 1B). Average volumes increased from 18.8 ± 6.8 mm^3^ at week 14 to 47.5 ± 15.9 mm^3^ at week 16. Despite growth heterogeneity between littermates, tumor volumes doubled within a 2-week period in all animals when tumor volumes reached 5-10 mm^3^. Therefore, preclinical testing of anti-cancer drugs was started about 14 weeks after transgene induction and when tumor volume was at least 5-10 mm^3^.

### Anti-cancer effects of Vandetanib are mediated by disruption of tumor vasculature

**Figure 2:**
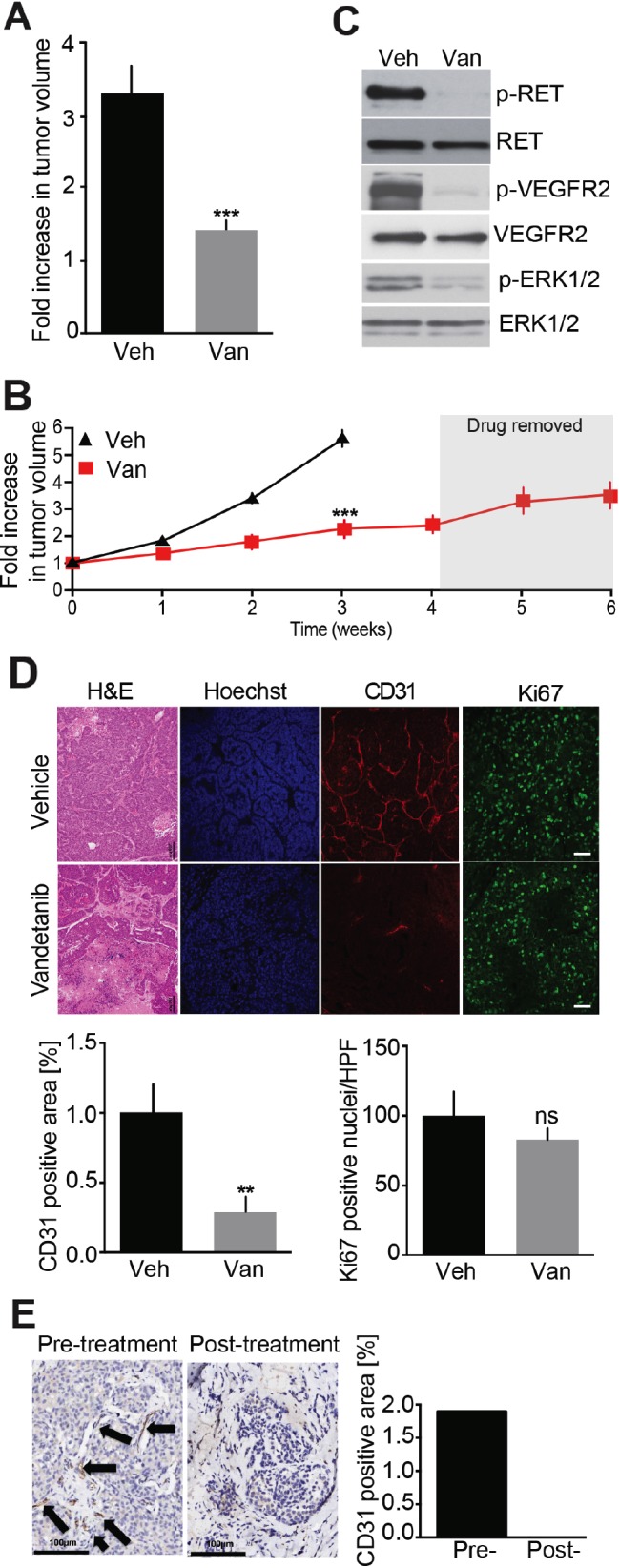
Evaluation of Vandetanib anti-tumor effect in a MTC mouse model **(A)** Analysis of tumor volumes after a 2-week treatment with either Vandetanib (25 mg/kg/day, Van, o.g.) (N = 5) or vehicle (Veh, o.g.) (N = 6). **(B)** Monitoring of tumor volumes by MRI during 6-weeks. Mice were dosed with Vandetanib (25 mg/kg/day, i.p.) (N = 4) or vehicle (i.p.) (N = 4) for 4 weeks and treatment was stopped for the next 2 weeks. **(C)** Immunoblot analysis of tumor lysates after a 3-week Vandetanib or vehicle treatment. (N = 3) **(D)** Immunohistological analysis of tumors for CD31 and Ki67 and quantifications after a 3-week Vandetanib or vehicle treatment. **(E)** CD31 staining and quantification of a MTC patient tumor biopsy before and after Vandetanib treatment. Scale bars are 100 μm. Arrows indicate microvessels.

As an important first step, Vandetanib, a drug currently in clinical use to treat MTC, was used to treat MTC model mice. Initial tumor volume was determined and either Vandetanib or control vehicle were administered for 2 weeks. Tumor volumes increased by 3.2-fold in the vehicle-treated animals during this period, whereas tumor volumes increased only by 1.2-fold in the Vandetanib-treated animals (Figure 2A). Subjects treated with vehicle alone exhibited severe signs of morbidity necessitating euthanasia. However, tumor progression remained minimal in animals subjected to an additional 2 weeks of Vandetanib treatment (4 weeks total, Figure 2B). Resumption of tumor growth was apparent upon discontinuation of Vandetanib administration.

We have previously demonstrated that mouse tumors and MTC patient samples were indistinguishable with regard to the activation of a number of oncogenic signaling pathways [16]. For example activated RET, a hallmark of MTC, is detected in mouse MTCs as evidenced by pY1062 RET immunoreactivity (Figure 2C). Also RET signaling was disrupted in Vandetanib-treated animals as evidenced by decreased phosphorylation of RET at Tyr-1062. Furthermore, VEGFR2 inactivation occurred, as determined by attenuation in phosphorylation of this receptor tyrosine kinase (RTK) at Tyr-1115. These effects were accompanied by reduced activating phosphorylation of ERK1/2. These effects were consistent with the established role of Vandetanib as a VEGFR2 and RET inhibitor [11]. These data demonstrate that the MTC mouse model is suitable to test and characterize the modes of action of Vandetanib as well as other RET inhibitors or alternative anti-cancer therapies.

Histological analysis of the tumors by H&E and Hoechst staining revealed numerous necrotic areas and reduced cellular density (Figure 2D). To better understand these effects, microvessel density was evaluated by staining CD31-positive endothelial cells within tumors. Microvessel density was reduced 3.5-fold in Vandetanib-treated animals compared to controls. These data indicate that Vandetanib invokes an anti-angiogenic response in MTC tumors as a major mode of action. This is consistent with the known spectrum of Vandetanib as a VEGFR2 inhibitor and the well-characterized role of that receptor in tumor angiogenesis.

Despite the anti-angiogenic effects revealed here, Vandetanib was initially developed as a RET inhibitor with anti-proliferative potential. To evaluate this mode of action, cell proliferation was assessed by Ki67 staining. Surprisingly, cell proliferation was unaffected by Vandetanib treatment compared to vehicle-treated controls (Figure 2D). Together these findings suggest that Vandetanib acts by disrupting tumor vasculature, resulting in tumor tissue necrosis, without directly affecting cell growth and total tumor volume.

To determine if this effect occurs in humans, we obtained very rare pathology samples from a single MTC patient biopsied before and after treatment with Vandetanib. Interestingly, microvessel density within these samples was reduced after treatment, which is similar to the effects observed in MTC model mice (Figure 2E). These results, albeit from a very rare single human case, support that Vandetanib may act primarily as an anti-angiogenic drug and, that our MTC mouse model is clinically relevant and can be used for the preclinical testing of anti-cancer therapies.

**Figure 3:**
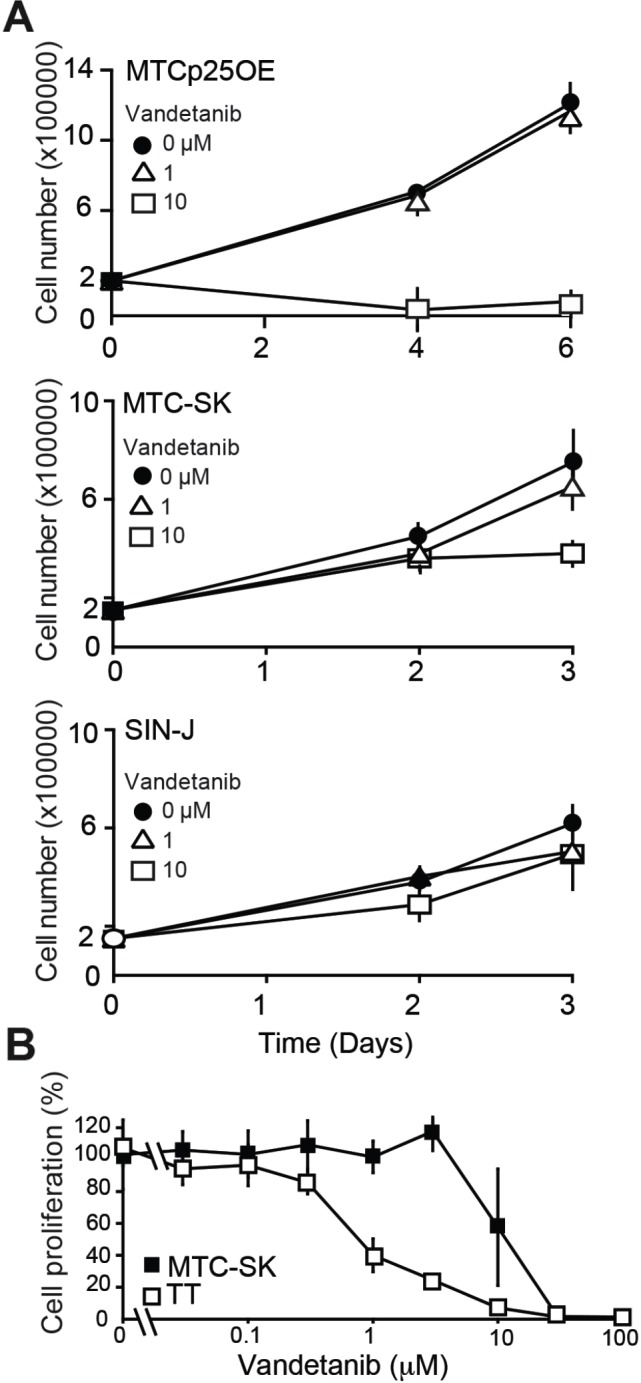
Analysis of Vandetanib anti-proliferative effect in cultured MTC cells **(A)** Proliferation assay of MTC cell lines treated with Vandetanib. **(B)** Cell survival assay and dose-response curve of TT cells and MTC-SK cells after a 6-day Vandetanib treatment. N = 2 experiments, N = 3 replicates/experiment.

Given the lack of effect of Vandetanib on cell proliferation in mouse tumors, we examined how Vandetanib impacted the proliferation of cultured MTC cells derived from our mouse model (MTCp25OE) [16] as well as two cell lines derived from non-RET mutated sporadic human MTC (MTC-SK and SIN-J) (Figure 3A) [17, 18]. Previous studies showed the proliferation of these human cell lines to be Cdk5-dependent [16]. MTCp25OE cells were not inhibited by 1 μM Vandetanib, although anti-proliferative effects were achieved at a concentration of 10 μM. Effects at both 1 and 10 μM were limited in both MTC-SK and SIN-J human sporadic MTC cell lines. These results further support that Vandetanib lacks strong anti-proliferative capacity toward sporadic forms of MTC, which depend upon aberrant Cdk5 activity for proliferation and do not harbor known RET mutations.

Mutations that constitutively activate RET are always detected in familial forms of MTC and Multiple Endocrine Neoplasia (MEN) 2A and the proliferation of MEN2 patient-derived MTC cell lines is inhibited by Vandetanib [19]. To determine if Vandetanib has stronger anti-proliferative effects in MTC cells harboring RET mutations, we compared the dose-dependent effects of Vandetanib on sporadic (MTC-SK) and familial (TT) MTC cell lines (Figure 3B). A concentration of 1 μM Vandetanib inhibited TT cell proliferation by >50% while it did not stop MTC-SK cell proliferation (Figure 3A, 3B). This observation indicates that Vandetanib exhibits greater potency toward RET-mutated TT cells compared to sporadic MTC-SK cells. This suggests that Vandetanib anti-proliferative properties are dependent on the RET mutation status in cultured MTC cell lines. However the anti-angiogenic properties of the drug may predominate over anti-proliferative effects *in vivo*, given that RET mutational status in MTC patients does not correlate with responsiveness to Vandetanib treatment [5].

### Evaluation of a second-generation TKI, Nintedanib as a treatment for MTC

**Figure 4:**
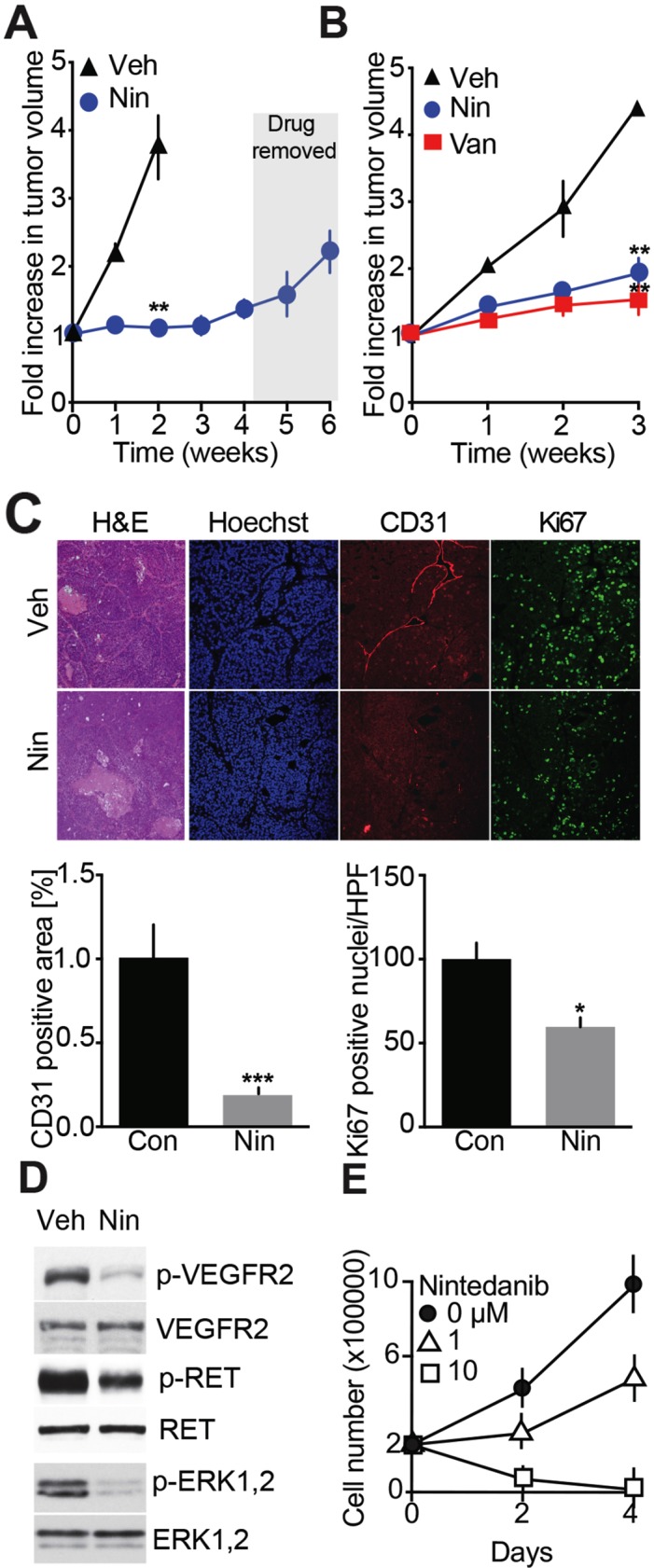
Evaluation of Nintedanib anti-MTC tumor and cell anti-proliferation effects **(A)** Effects on tumor volume monitored by MRI over 6-weeks. Mice were dosed with Nintedanib (35 mg/kg/day, i.p., Nin) (N = 3) or vehicle for 4 weeks (N = 3) and treatment was stopped for the next 2 weeks. **(B)** MRI-monitoring of tumor growth during a 3-week treatment with either Vandetanib (25 mg/kg/day, o.g.) (N = 4), Nintedanib (100 mg/kg/day, o.g.) (N = 4) or vehicle (N = 3). (^**^p=0.0031, one-way ANOVA, Bonferroni's post hoc, week 3) **(C)** Immunohistological analysis of tumors for CD31 and Ki67 and quantifications after a 3-week Nintedanib or vehicle treatment. Scale bars are 100 μm, (N = 4). **(D)** Immunoblot analysis of tumors after a 3-week treatment with Nintedanib or vehicle (N = 3). **(E)** Proliferation assay of MTC cell lines treated with Nintedanib. N = 2 experiments, N = 3 replicates/experiment.

Having found that Vandetanib acts via anti-angiogenesis and to expand the repertory of drugs available for MTC patient treatment, we decided to test a TKI with proven anti-angiogenic properties. Nintedanib antagonizes RET and VEGF receptors and blocks endothelial cell proliferation and tumor growth in mouse models of pancreatic and lung cancers [15, 20]. In our MTC animal model, Nintedanib arrested tumor growth across a 4-week treatment period compared to vehicle-treated controls (Figure 4A). Furthermore, Nintedanib showed the same tumor progression inhibition capability as Vandetanib across 3 weeks of treatment (Figure 4B). To determine if the Nintedanib inhibitory effect was dependent on continuous drug administration, treatment was interrupted for 2 weeks (Figure 4A). As previously seen for Vandetanib, tumors resumed growth once Nintedanib treatment was discontinued. Histological examination of tumors after a 3-week Nintedanib regimen revealed necrosis, tissue disorganization and a 5-fold reduction in CD31-positive cells (Figure 4C). Thus the anti-angiogenic effect of Nintedanib was greater than that of Vandetanib (Figure 2D) and was accompanied by a statistical significant attenuation in cancer cell proliferation as evidenced by a 1.5-fold reduction in Ki67 signal (Figure 4C). Consistent with its anti-angiogenic effects, Nintedanib therapy also reduced phosphorylation of RET, VEGFR2, and ERK1/2 in MTC tumors (Figure 4D).

To further characterize the anti-proliferative effect of Nintedanib, MTCp25OE cells were treated with the drug for 2-4 days. Cell proliferation was inhibited by 1 μM Nintedanib while 10 μM drug caused cell death (Figure 4E), thereby confirming that Nintedanib exerts anti-proliferative effect on cancer cells. As a second set of experiments, the effects of Nintedanib on RET-mutated familial MTC-derived TT cell growth was compared to that of Vandetanib. In dose-response analysis, Nintedanib exhibited 10-fold greater anti-proliferation potency than Vandetanib (Supplementary Figure 1) with IC_50_ values (±SEM) of 0.12 (±0.05) μM for Nindetanib and 1.09 (±0.28) μM for Vandetanib. Thus Nintedanib has stronger anti-proliferative properties than Vandetanib in sporadic and RET-mutated familial cultured MTC cells and in our MTC mouse model. Overall our observations indicate that Nintedanib may have stronger anti-cancer effects than Vandetanib but with no improved outcome on decreasing tumor volume, which suggests that Nintedanib and Vandetanib may have distinct mechanisms of action.

### Preclinical evaluation of a combination TKI/HDAC inhibitor therapy for MTC

**Figure 5:**
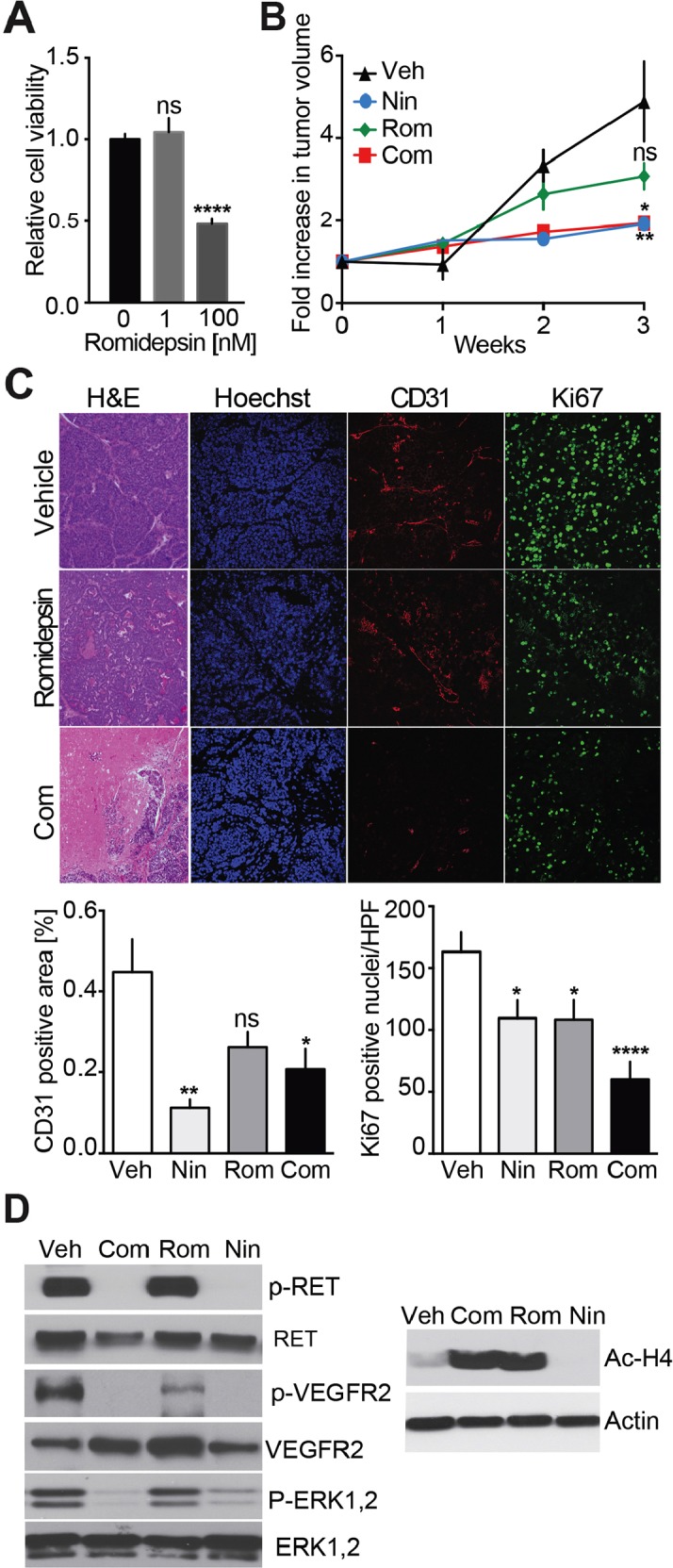
Analysis of Nintedanib/Romidepsin anti-tumor effect on MTC proliferation **(A)** Cell survival analysis of MTCp25OE following treatment with increasing Romidepsin concentrations (^****^p<0.0001, one-way ANOVA, Bonferroni's post hoc) **(B)** Monitoring of tumor volumes by MRI during a 3-week treatment with Nintedanib (35 mg/kg/day i.p.) (N = 5), Romidepsin (0.78 mg/kg/day, i.p.) (N = 5), or Nintedanib (35 mg/kg/day, i.p.) + Romidepsin (0.38 mg/kg/day, i.p., Com) (N = 10) or vehicle (i.p.) (N = 11) (^***^p<0.005, one-way ANOVA, Bonferroni's post hoc, week 3). **(C)** Immunohistological analysis of tumors for CD31 and Ki67 and quantifications after a 3-week treatment with either Romidepsin or Nintedanib + Romidepsin (Rom), or vehicle. Scale bars are 100 μm. **(D)** Immunoblot analysis of tumors after a 3-week treatment with vehicle, Nintedanib + Romidepsin, Romidepsin, or Nintedanib (N = 3).

Both Nintedanib and Vandetanib have strong anti-angiogenic effects *in vivo* while exhibiting no to moderate anti-proliferation properties. HDAC inhibitors are considered promising anti-mitotic agents, which may render solid tumors more vulnerable to other anti-cancer therapies. Romidepsin is an FDA approved HDAC inhibitor which exhibited synergistic inhibitory effects with TKIs in the treatment of non-small cell lung cancer (NSCLC) in preclinical models [21–23] and in clinical trials [24]. We tested the anti-proliferative effect of Romidepsin on mouse MTC cells in culture. A 50% reduction in cell growth was achieved at a concentration of 100 nM (Figure 5A). We then evaluated the anti-tumor effects of Romidepsin alone and in combination with Nintedanib *in vivo*. Romidepsin treatment exhibited a trend toward reduction of MTC tumor growth (Figure 5B). Combinatorial treatment with Romidepsin and Nintedanib slowed tumor growth but showed no improved effect on measured tumor volume compared to Nintedanib alone. Romidepsin reduced Ki67 values by 1.5-fold (Figure 5C), which was comparable to Nintedanib effect on Ki67 values. Importantly, the combination caused a 2.7-fold decrease in this marker of cell proliferation, thereby showing a synergistic effect of combined treatments on cell proliferation. However, Romidepsin did not significantly reduce microvessel density while Nintedanib alone or in combination lowered CD31 staining.

Romidepsin had no effect on phosphorylation of RET, VEGFR2, or ERK1/2 while Nintedanib alone or in combination with Romidepsin lowered these signals, consistent with the previous results (Figure 5D). Importantly, the level of de-acetylated histone H4 was increased in tumors from Romidepsin and Romidepsin + Nintedanib treated animal but not in those treated with Nintedanib alone. These observations confirm that Romidepsin blocks HDAC activity as expected but not RTK signaling, while the combination of Romidepsin and Nintedanib blocked both HDAC and RTK signaling. While Romidepsin and Nintedanib synergized mechanistically, the combination therapy did not decrease tumor volume more efficiently than Nintedanib alone.

To better understand the relationship between drug administration and tumor response from the perspective of intermittent treatments, such as those experienced by patients, MTC model mice were treated for extended periods with cycles of drug treatment and removal (Supplementary Figure 2). After 3 cycles, tumors appeared to achieve resistance to the drug. Together with the other results presented here, these observations indicate that chronic Nintedanib treatment is necessary to block tumor growth but that tumors may eventually become resistant to the drug.

### Nintedanib and Vandetanib inhibit PI3K/AKT signaling

**Figure 6:**
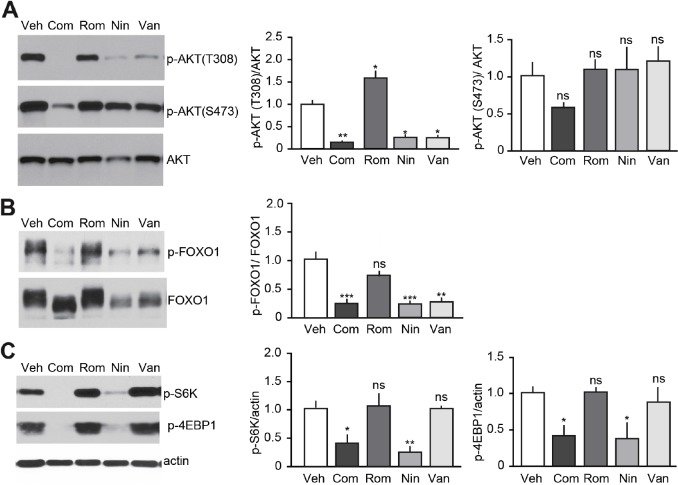
Effect of TKI-based therapies on the PI3K/AKT pathway Immunoblot analysis of tumors after a 3-week treatment with either Vandetanib (25 mg/kg/day), Nintedanib (35 mg/kg/day), Romidepsin (0.78 mg/kg/day) or Nintedanib (35 mg/kg/day) + Romidepsin (0.38 mg/kg/day, Com) or vehicle for **(A)** AKT, **(B)** FOXO1 and **(C)** mTOR, (N = 3-4) (p-AKT(T308), ^****^p<0.0001; p-AKT(S473), ns p=0.1433; p-FOXO1, ^***^p<0.005; p=S6K, ^***^p<0.005; p-4EBP1, ^***^p<0.005, one-way ANOVA, Bonferroni's post hoc).

Vandetanib and Nintedanib antagonize RET signaling, block tumor development but exhibit different anti-proliferative effects on tumor cells. These observations suggest that the mode of action of Vandetanib and Nintedanib are not identical and mediated not only via RET-dependent but also via RET-independent signaling pathways. The PI3K/AKT pathway is commonly deregulated in thyroid cancers, including MTC [25, 26]. Therefore, we examine how TKI- and HDAC-based treatments affect PI3K/AKT signaling cascades (Figure 6). AKT phosphorylation at Thr308 (phospho-Thr308) and Ser473 (phospho-Ser473) were detected in all mouse MTC tumors, demonstrating that the PI3K/AKT pathway is activated in these tumors, as observed in MTC patients [27, 28] (Figure 6A). Phospho-Thr308 AKT was reduced in tumors from Vandetanib, Nintedanib, and Nintedanib + Romidepsin treated mice, but not in mice treated with Romidepsin alone in comparison with vehicle-treated MTC mice. Phospho-Ser473 remained unchanged. These findings indicate that TKI-based treatments inhibited the PI3K/AKT pathway, and support the observation that phospho-Thr308 is a better marker of AKT activity than phospho-Ser473 [28]. Consistent with these effects, phosphorylation of FOXO1, an AKT downstream target, was reduced upon treatment with TKIs but not Romidepsin alone (Figure 6B). To investigate further the effect of TKI-based treatments on PI3K/AKT pathways, we examined the activation state of the mTOR signaling cascade, which is another well-characterized PI3K/AKT downstream target (Figure 6C). Nintedanib alone or in combination with Romidepsin inhibited mTOR signaling as evidenced by a decrease in the phosphorylation of mTOR downstream effectors, S6K and 4EBP-1. Interestingly, Vandetanib had no effects on mTOR. Together these findings indicate that Vandetanib and Nintedanib use different mechanisms downstream of PI3K/AKT to stop MTC growth, although the two TKIs show several mechanistic similarities. These data also suggest that mTOR targeting is dependent on the type of TKI that is administered.

## DISCUSSION

Here we conducted *in vivo* studies using a unique animal model of MTC to examine the anti-cancer effects of Vandetanib, one of only two drugs approved for MTC therapy. Biopsies and histological evaluations are rarely performed during or following chemotherapy treatment, and surprisingly few preclinical studies have examined the mode of action of TKIs in MTC. The MTC mouse model used here encompasses molecular and physical features related to sporadic MTC patients, which represent 75% of all MTC cases. The model exhibited responses to Vandetanib and histological effects consistent with those observed in human patients. Thus our mouse model of MTC appears valid for the preclinical testing of drug treatments for MTC. Investigations with this model could provide insight regarding the modes of action, doses, and administration schedules prior to testing in cancer patients [29].

The impetus behind Vandetanib use as a MTC therapy stemmed from its inhibition profile which included antagonization of RET, the oncogene product often regarded as the main MTC driver [1, 11, 30]. Here we found that Vandetanib stopped tumor growth *in vivo* by causing necrosis, while having no effect on MTC cell proliferation. This observation indicates that the mode of action of Vandetanib is primarily through tumor associated-endothelial cells and subsequent blood vasculature reduction rather than via direct inhibition of RET in MTC tumor cells, as previously observed in preclinical model of colon cancers [29]. Likewise, the RET-targeting TKI, Sunitinib, mediates its renal cell carcinoma anti-tumor effect via tumor-associated endothelial cells and not tumor cells [31]. In neuroblastoma, Vandetanib exerts a dual action by targeting VEGF receptors on tumor endothelial cells and RET on tumor cells as demonstrated in neuroblastoma [32]. Together with our observations, these studies shows that TKIs do not always directly target tumor cells as may be expected from cell culture studies, but instead may target either the tumor microenvironement, tumor cells, or both. Characterizing the microenvironment of MTC tumors may help define markers and thus stratify patients so that those likely to benefit from Vandetanib treatment may be identified. It would also be of interest to determine if the recently developed and very specific RET inhibitors, BLU-667 and LOXO-292 [4] exhibit improved anti-angiogenic and antiproliferative properties compared to Vandetanib and Nintedanib *in vivo* and in cell culture conditions.

The PI3K/AKT pathway has emerged as a valid cancer drug target. In particular, targeting the PI3K/AKT/mTOR pathways using mTOR inhibitors improved progression-free survival in patients with advanced pancreatic neuroendocrine tumors [33]. Here we show that Vandetanib inhibits the PI3K/AKT/FOXO1 pathway but not the PI3K/AKT/mTOR pathway in MTC mouse tumors. These observations support the rationale of combining Vandetanib and an mTOR inhibitor to improve MTC treatment, an approach that is underway in current clinical trials (see https://clinicaltrials.gov/ Identifier NCT01582191). Preclinical studies in colon cancer mouse models have demonstrated that the doses and administration schedules (*e.g.*, sequential or concurrent) of Vandetanib or drug combination could change the treatment outcome [13, 29]. Furthermore, Nintedanib, which blocks VEGFR2, RET, ERK1,2 and PI3K/AKT/FOXO1 like Vandetanib, also inhibits PI3K/AKT/mTOR, but may still have limited long-term anti-tumor effects on MTC due to the development of resistance. Therefore determining the right doses and best schedule may be crucial for the success of a Vandetanib/mTOR inhibitor clinical trial. It may also be important to determine if a Vandetanib/mTOR inhibitor combination or a Nintedanib monotherapy is the most beneficial through future patient-centered studies. Regardless, targeted therapies other than TKIs that affects these pathways should be explored further. For example, the inhibitor of the molecular chaperone HSP90 (AUY922) targets RET and mTOR signaling and inhibits the growth of cultured MTC cell lines [34].

While Vandetanib and Nintedanib both stopped tumor development, they had weaker effects than expected on tumor cell proliferation based on previously published studies [19]. We hypothesized that combining these TKIs with an antiproliferative drug could improve TKI efficacy on MTC mouse tumor development. We chose the cyclic HDAC inhibitor Romidepsin to test in combination with Nintedanib. Although tumor cell proliferation and microvessel density were decreased, synergesitic effects of the two drugs *in vivo* did not occur under our experimental conditions. In fact the overall effect on tumor growth was similar to that observed for Nintedanib alone. Although HDACs have shown promise as combinatorial treatment components, they bring the added complication of potentially problematic side effects at the doses that may be required for efficacy when used in the context of other drug burdens.

The TKIs tested here caused histological changes in the tumors with large necrotic areas arising. However a survey of apoptotic markers in our mouse tumors yielded negative results (data not shown). This did not exclude necroptosis as an additional mode of action in these tumors. Our data suggests that the use of Nintedanib in combination with Romidepsin for MTC is unwarranted. However, other cytotoxic drugs might be combined with Vandetanib or Nintedanib administration. For example, irritonican was used successfully in preclinical models of colon cancer [29]. Also, Aurora kinase inhibitors have shown strong anti-tumor effects in several types of cancer [35, 36]. Testing these drugs preclinically in our animal model will provide future insight into the potency of these combinations.

The limited effects of Vandetanib and Nintedanib on sporadic MTC cell proliferation suggest that targeted therapies other than TKIs should be further explored. Given the role of Cdk5 in MTC proliferation [16] and in angiogenesis [37], Cdk5 inhibitors might be considered as therapeutic drugs to be used alone or in combination with a TKI such as Nintedanib. In recent years, numerous therapies, including antibody-drug conjugates, immune checkpoint therapies and peptide receptor radionuclide therapy, which have potential applications for MTC treatment [4] have been developed. Some have been approved by the FDA for other clinical applications than MTC. Given the lack of therapeutic options available for MTC patients it will be important to determine if these drugs can be re-purposed and fast-track approved for MTC therapy.

## MATERIALS AND METHODS

### Mouse line

NSE-tTA/tetOp-p25-GFP bi-transgenic mice were derived by crossing NSE-tTA and the tetOp-p25-GFP mouse lines as previously described [16]. In this system, p25-GFP expression is controlled by a tetracycline response element (TRE), which is activated by binding of a tetracycline transactivator (tTA). In presence of doxycycline (Dox), tTA binds to Dox instead of the TRE. Dox (100 mg/ml) is added to drinking water until weaning. Tumor growth is induced by removing Dox. All procedures were approved by the Institutional Animal Care and Use Committees of the University of Texas and University of Alabama Birmingham conducted in accordance with the applicable portions of the National Institute of Health Guide for the Care and Use of Laboratory Animals.

### Human samples

Lymph node MTC metastasis were obtained after written informed consent from the patient was obtained.

### Drugs

Vandetanib (#V-9402) and Nintedanib (#N-9077) were obtained from LC Laboratories. Romidepsin (#3515) was purchased from Tocris R&D.

### Animal testing

Unless stated otherwise, drugs were administered by intra-peritoneal (i.p.) injections. Drug doses were determined based on published studies [38–40] (see also Supplementary Figure 3) and were as indicated in the Figure legends. Due to adverse events, Romidepsin was used at 0.78 mg/kg/day instead of 1.2 mg/kg/day [21] for monotherapy. Vandetanib and Nintedanib were administered initially by oral gavage (o.g.) 6 days/week for experiments lasting up to 3 weeks. TKI administration was switched to i.p. injections 6 days/week when treatment protocols were over 3-weeks. Before switching from o.g. to i.p. administration, we confirmed that the doses administered using the different routes led to comparable antitumor effects (Supplementary Figure 3). Romidepsin was always administered i.p. twice a week. For combinatorial treatment, Romidepsin was injected i.p. twice a week and Nintedanib 3 times a week. Romidepsin and Nintedanib were not injected on the same day. No drug was administered on the day that mice were imaged. Vandetanib and Nintedanib were solubilized in citrate buffer pH 6.2 and pH 4, respectively. Romidepsin was dissolved in DMSO and diluted in PBS.

### Magnetic resonance imaging

All MRI studies were conducted in a 7T small-animal scanner with a 38-mm inner diameter radio frequency (RF) coil (Agilent, Palo Alto, Calif). Under anesthesia with 1-2% isoflurane (Aerrane; Baxter Health Care, Deerfield, Ill) mixed in 100% oxygen, all animals were placed supine with the thyroid centered with respect to the center of the RF coil. Following initial localization, high-resolution axial T2W images were obtained to cover the entire thyroid with a fast spin-echo sequence. The imaging parameters were: repetition time = 2500 ms, effective echo time = 40 ms, field of view = 32^2^ mm, matrix size = 256^2^ (125 μm in-plane resolution), slice thickness = 1 mm, gapless, number of excitations = 8, affording a total scan time of 10 min 45 s. For analysis, the entire volume of the tumor was measured by means of manual segmentation on the T2W image in all slices. All image processing was conducted using an imaging processing software (ImageJ, version 1.50i).

### Tissue collection

At the end of treatments, or if animals were in distress, animals were culled by asphyxia using CO_2_ and perfused using PBS containing protease and phosphatase inhibitors as described [16]. One side of the tumor was fixed in 4% paraformaldehyde (PFA) for 48 h prior to paraffin embedding and the other side was frozen in dry-ice upon collection.

### Immunoblotting

Quantitative immunoblot analyses were conducted using standard procedures [16]. Antibodies directed to ERK1/2 (#9102), phospho-ERK1/2 (#9101), VEGFR2 (#9698), phospho-VEGFR2 Tyr1115 (#3770), acetyl-histone H4 Lys8 (#2594), phospho-AKT Thr308 (#4056), phospho-AKT Ser473 (#3787), AKT (#4691), phospho-FOXO1 Ser256 (#9461), FOXO1 (#2880), phospho-4EBP1 Thr37/46 (#2855), phospho-S6K Ser235/236 (#4858) were purchased from Cell Signaling Technology and used at dilutions of 1:1000. Antibodies to RET (# ab134100) and phospho-RET Tyr 1062 (#ab51103) were purchased from Abcam and used at dilutions of 1:10,000 and 1:2000 respectively. Antibodies to Actin (#A2228) were from Sigma-Aldrich and used at dilution 1:10,000.

### Immunostaining

Rat monoclonal CD31 (PECAM-1), Clone SZ31 (DIA-310) antibodies were from Dianova and rabbit polyclonal Ki-67 antibodies from Abcam (ab15580). Paraffin sections were boiled in citrate buffer pH 6.0 for antigen unmasking. Primary antibodies were used at a dilution of 1:100. Alexa Fluor®546 conjugated goat anti-rat secondary antibodies (for CD31) and Alexa Fluor®647 conjugated chicken anti-rabbit secondary antibodies (for KI-67) were from Life Technologies and used at a dilution of 1:400. Sections were counterstained with Hoechst 33342 (5 mg/ml) for nuclear detection. Images were obtained with a Leica SP8SMD laser scanning confocal microscope using a 40x oil immersion lens, λ 561 nm and λ 563 nm laser lines for excitation and emission between 570 and 620 nm, and 650 and 700 nm, respectively. There was no overlap with endogenous p25-EGFP signal from the bitransgenic animal. The 633 nm channel was pseudo-colored in green, 561 nm channel in red and Hoechst in blue. For each conditions, N=3 fields from N=4 animals were imaged. Image analyses were conducted using Fiji17. Values reported are %CD31 staining and Ki67 positive cells /field of view.

### Cell culture, proliferation and survival assays

Mouse and human MTC cell lines, *i.e.* MTCp25OE, MTC-SK, SIN-J and TT cells have been previously described [16, 41, 42]. Briefly, MTC-SK cells were derived from sporadic patient primary tumor [43], SIN-J cells from a sporadic MTC patient lymph node metastasis [41]. TT cells were from an hereditary MTC patient needle biopsy [42]. Cells were maintained and proliferation assays were conducted using the WST-1 cell proliferation reagent (Roche Diagnostics) [16]. For cell survival assays and dose-response curves, cells were seeded onto 96-well TC plates, treated with Vandetanib or Nintedanib for 6 days, fed with 10% FBS media on day 3, then incubated with CyQuant Cell Proliferation Assay (Invitrogen) reagents per manufactor's protocol and assayed using a BMG Optima Fluostar microplate reader. RFU from each well was normalized to control treated cells in order to determine percent effect on survival.

### Statistical analysis

All quantitative data are expressed as means ± S.E.M. Two-tailed, unpaired Student's *t*-test or one-way ANOVA followed by a Bonferroni's post-test were used to compare the different groups as needed. Probability values of 0.05 or lower were considered to indicate significant differences between groups. The symbols used to indicate p-values were ^*^p < 0.05, ^**^p < 0.01, ^***^p < 0.005, with subject number (n) stated in the legend.

## SUPPLEMENTARY MATERIALS FIGURES



## Abbreviations

MTC: Medullary Thyroid Carcinoma
NE: Neuroendocrine
TKI: Tyrosine Kinase Inhibitor
RTK: Receptor Tyrosine Kinase
MRI: Magnetic Resonance Imaging
