# Supplementary material for: Preclinical characterization of tyrosine kinase inhibitor-based targeted therapies for neuroendocrine thyroid cancer
Source: Oncotarget. 2018 Dec 28;9(102):37662–75. doi: 10.18632/oncotarget.26480 (PMC6340867; doi:10.18632/oncotarget.26480)
Supplement: Supplementary file 1 [file oncotarget-09-37662-s001.pdf]

Preclinical characterization of tyrosine kinase inhibitor-based targeted therapies for neuroendocrine thyroid cancer

SUPPLEMENTARY MATERIALS

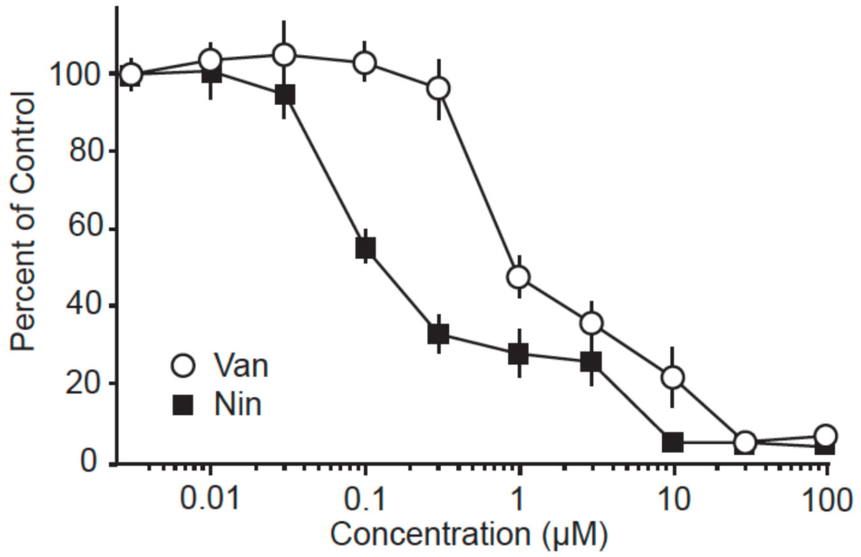

Supplementary Figure 1: Dose-response curves for treatment of TT cells after a 6-day treatment with either Vandetanib or Nintedanib.

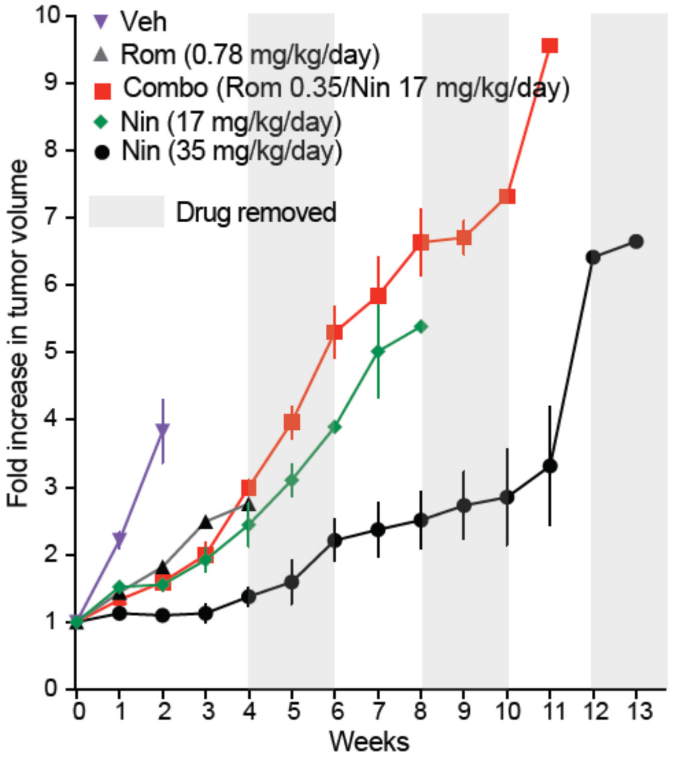

Supplementary Figure 2: Long-term anti-tumor effects of Nintedanib/Romidepsin combination therapy. Monitoring of tumor volumes by MRI during 13-weeks. Mice were dosed as indicated for 4 weeks, treatment was stopped for the next 2 weeks (Grey area), then restarted for 2 more weeks, and repeated. A total of 2-3 drug administration/drug arrest cycles were done. Data for weeks 1-3 is the same as in Figure 5B.

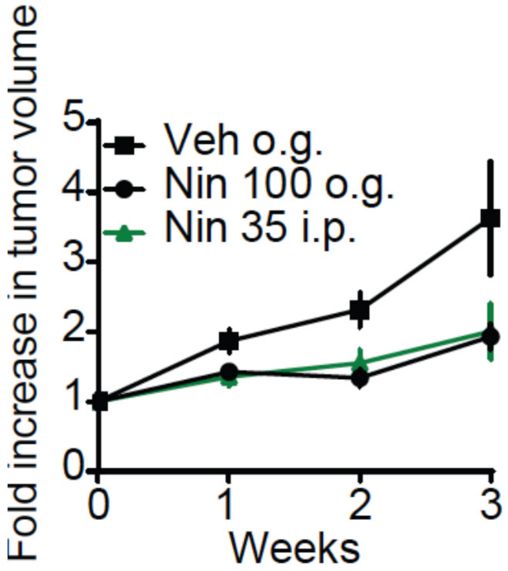

Supplementary Figure 3: Effect of drug concentration and delivery on tumor volume. Monitoring of tumor volumes by MRI during 3-weeks. Mice were dosed with either Nintedanib 100 mg/kg/day by oral gavage (o.g.) (Nin 100 o.g.) (N = 8 mice) or with Nintedanib 35 mg/kg/day by intra-peritoneal injection (i.p.) (Nin 35 i.p.) (N = 4 mice) or vehicle (N = 8, o.g.) for 3 weeks and the effect of each drug/dose on tumor growth was compared.
